# Meeting Abstracts from the International Burden of Disease Conference 2022: Belgrade, Serbia.

**DOI:** 10.1186/s13690-023-01129-9

**Published:** 2023-07-12

## A1 The Burden of mental disorders in Iran, 1990-2019: results from the global burden of disease study 2019

### Jalal Arabloo^1^, Samad Azari^2^

#### ^1^Health Management and Economics Research Center, Health Management Research Institute, Iran University of Medical Sciences, Tehran, Iran; ^2^Hospital Management Research Center, Health Management Research Institute, Iran University of Medical Sciences, Tehran, Iran

##### **Correspondence:** Jalal Arabloo (arabloo_j64@yahoo.com)

*Archives of Public Health 2023*, **81(Suppl 2):**A1

**Background**

Mental disorders are increasing in Iran. A systematic analysis of the disease burden provides the basis for targeted health policies on mental health in Iran.

**Methods**

We used GBD 2019 study data [1,2] to estimate the incidence, prevalence, years lived with disability (YLDs), years of life lost (YLLs) and disability-adjusted life-years (DALYs) from mental disorders from 1990 to 2019 in Iran. The mental disorders included in the GBD 2019 were depressive disorders, anxiety disorders, bipolar disorder, schizophrenia, autism spectrum disorders, conduct disorder, attention-deficit hyperactivity disorder, eating disorders, idiopathic developmental intellectual disability, and other mental disorders. We reported rates per 100,000 population, percentage changes in 1990-2019, and 95% Uncertainty Intervals (UIs) for the estimates. All data related to this abstract are available at GBD Results tool [https://vizhub.healthdata.org/gbd-results] [3].

**Results**

In 2019, 6.7 [95% uncertainty interval (UI): 5.8-7.8] million incident cases of mental disorders in Iran, 15.7 million (95% UI: 14.5-16.9) prevalent cases and 2.05 (95% UI: 1.5-2.7) million DALYs were estimated. Between 1990 and 2019, the number of DALYs due to mental disorders increased from 1.1 million (UI: 0.85–1.5) to 2.05 (95% UI: 1.5-2.7). Age-standardized DALY rates increased 1.8% between 1990 [2,254 (95% UI 1,670–2,965) per 100 000 population] and 2019 [2295.8 (95% UI 1702.2–3033.6) per 100 000 population]. The age-standardized DALY rate was 1,976.27 (95% UI 1,455.52– 2,599.31) among males and 2,623.57 (95% UI 1,927.49– 3,470.32) among females in 2019. Depressive disorders was the leading cause of burden with 890.32(95% UI 605.75– 1,247.77) age-standardized DALY rate.

**Conclusions**

Strategies and providing appropriate mental health services for those with mental health disorders is necessary to combat their increasing burden. The COVID-19 pandemic has also adversely affected mental health. Policy change is needed to address the impact of COVID-19 on Iranian Mental Health [4].

**Acknowledgements**

We acknowledge the Institute for Health Metrics and Evaluation at the University of Washington for creating the opportunity to use the GBD database.

**References**

1. Murray CJL, Aravkin AY, Zheng P, et al. Global burden of 87 risk factors in 204 countries and territories, 1990-2019: a systematic analysis for the Global Burden of Disease Study 2019. Lancet. 2020; 396: 1223-1249.
2. Vos T, Lim SS, Abbafati C, et al. Global burden of 369 diseases and injuries in 204 countries and territories, 1990-2019: a systematic analysis for the Global Burden of Disease Study 2019. Lancet. 2020; 396: 1204-1222.
3. GBD Results tool: Global Burden of Disease Collaborative Network. Global Burden of Disease Study 2019 (GBD 2019) Results. Seattle, United States: Institute for Health Metrics and Evaluation (IHME), 2020. Available from https://vizhub.healthdata.org/gbd-results/.
4. Doshmangir L, Gholipour K, Gordeev VS. Policy changes needed to address the mental health impact of COVID-19 in Iran. Lancet Psychiat. 2022 ;9(8):e35.

## A2 Burden of lung cancer associated with occupational exposure to hexavalent chromium

### José Chen-Xu^1,2^, Lea Sletting Jakobsen^3^, Sara Monteiro Pires^3^, Susana Viegas^1^

#### ^1^National School of Public Health, Public Health Research Centre & Comprehensive Health Research Centre, Universidade NOVA de Lisboa, Lisbon, Portugal; ^2^Public Health Unit, Primary Healthcare Cluster Baixo Mondego, Coimbra, Portugal; ^3^National Food Institute, Technical University of Denmark, Lyngby, Denmark

##### **Correspondence:** José Chen-Xu (josechenx@gmail.com; jc.xu@ensp.unl.pt)

*Archives of Public Health 2023*, **81(Suppl 2):**A2

**Background**

Exposure to hexavalent chromium [Cr(VI)] occurs in several occupational activities, including welding and electroplating. This still occurs widely at EU level, and it has been associated with lung cancer. The occupational exposure limit is set to change to 5 μg/m^3^ starting from 2025, however current limits are higher. This remained unchanged with the 2022 European Directive revision. This study aims at assessing the burden of lung cancer caused by occupational exposure to Cr(VI).

**Methods**

Data were extracted from Global Burden of Disease 2019 study, Eurostat and relavant literature. Estimates were made of the number of cases of cancer attributable to workplace exposure to Cr(VI) and related DALYs, with and without more stringent exposure limit levels.

**Results**

With current exposure limite values (10 μg/m^3^ and 25 μg/m^3^ for welding), 270 cases of lung cancer in EU in 2019 would be attributed to Cr(VI) exposure, resulting in 4,942 DALYs. If the welding industry adopted the 10 μg/m^3^ limit, the burden would be 42 cases and 769 DALYs. By applying the limit of 5 μg/m^3^ foreseen for 2025, it is predicted a decrease of 156 cases and 2,855 DALYs, still causing 114 lung cancer cases and 2,086 DALYs. Lower limits would further prevent cases, with a 0.5 μg/m^3^ limit preventing 259 cases, corresponding to 4,733 DALYs.

**Conclusion**

Drawing scenarios with different limits of exposure to Cr(VI) allowed to understand the impact of EU regulatory policies in occupational health, making a strong case for adapting more protective exposure limits to prevent occupational cancer cases and reduce DALYs and associated costs.

**Acknowledgements**

The authors would like to thank the researchers from the Research Group for Risk Benefit from the National Food Institute, Technical University of Denmark for raising questions to improve the robustness of the research project, and the COST Action CA18218 European Burden of Disease Network, for supporting with the Short-Term Scientific Mission.

## A3 Years of life lost to COVID-19 in the Federation of Bosnia and Herzegovina during 2020-2021

### Šeila Cilović Lagarija, Siniša Skočibušić

#### Institute for public health Federations of Bosnia and Herzegovina, Sarajevo, Bosnia and Hercegovina

##### **Correspondence:** Šeila Cilović Lagarija (s.cilovic@zzjzfbih.ba)

*Archives of Public Health 2023*, **81(Suppl 2):**A3

**Background**

The full health impact of the COVID-19 pandemic is critical for evaluating the potential policy responses. We have analyzed the impact of COVID-19 on premature mortality by calculating the Years of Life Lost (YLL) in the Federation of Bosnia and Herzegovina in 2020-2021.

**Methods**

YLLs are calculated by subtracting the age at death from the longest possible life expectancy for a person at that age. For calculation YYL used the number of COVID-19 deaths, obtained from the Institute for Statistics of FBiH [1,2], multiplied by the life expectancy from Global Burden of Disease (GBD 2019), this allows comparisons by age groups as well as with other countries, and population size used to calculate YLL per 100 000 [3].

**Results**

During the year 2020 39,511 years of life was lost by men, whereas women lost 19,910 years of life, and a total of 59,420 YLL, 2,720 YLL per 100 000 (total rate). In the year 2021 men lost 72,503 years of life, women lost 54,582 years of life, which is a total of 127,085 YLL, 5,860 YLL per 100 000 (total rate). In comparison with other results form studies in 81 countries, a total of 20,507,518 years of life have been lost to COVID-19, due to 1,279,866 deaths from the disease [4].

**Conclusion**

From a public health standpoint, years of life lost is crucial as it assesses how much life has been cut short for populations that have been affected by the disease [3]. YLL is very closely associated with COVID-19 deaths in the country, and during the pandemic period, men lost 1.5 more YLL in comparison to women in the Federations of BiH.

**References**

1. Institute for Public Health FB&H. Health statistics annual FB&H 2021. Sarajevo, 2022
2. Institute for Public Health FB&H. Health statistics annual FB&H 2020. Sarajevo, 2021
3. Rommel A, Lippe EV, Plass D, Ziese T, Diercke M, Heiden MA, Haller S, Wengler A; BURDEN 2020 Study Group. The COVID-19 Disease Burden in Germany in 2020—Years of Life Lost to Death and Disease Over the Course of the Pandemic. Dtsch Arztebl Int. 2021 Mar 5;118(9):145-151. doi: 10.3238/arztebl.m2021.0147. PMID: 33958032; PMCID: PMC8212397.
4. Pifarré i Arolas, H., Acosta, E., López-Casasnovas, G. et al. Years of life lost to COVID-19 in 81 countries. Sci Rep 11, 3504 (2021). 10.1038/s41598-021-83040-3

## A4 The disease burden of COVID-19 in Belgium during the year 2020 and 2021

### Robby De Pauw^1,2^, Pierre Smith^1^, Lander Willems^3^, Niel Hens^3^, Jure Jurčević^1^, Brecht Devleesschauwer^1,4^

#### ^1^Department of Epidemiology and Public Health, Sciensano, Brussels, Belgium; ^2^Department of Rehabilitation Sciences, Ghent University, Ghent, Belgium; ^3^Department of Family Medicine and Population Health, University of Antwerp, Antwerp, Belgium; ^4^Department of Translational Physiology, Infectiology and Public Health, Ghent University, Merelbeke, Belgium

##### **Correspondence:** Robby De Pauw (Robby.DePauw@Sciensano.be)

*Archives of Public Health 2023*, **81(Suppl 2):**A4

**Background**

Since the outbreak of the severe acute respiratory syndrome coronavirus 2 in 2020, over 4 million cases of the virus-induced COVID-19 disease have been reported in Belgium. Researchers have world-wide tried to assess the impact of COVID-19 on the population health by estimating its disease burden in terms of disability-adjusted life years (DALYs). In this study, we have evaluated the DALYs due to COVID-19 in Belgium during the year 2020 and 2021.

**Methods**

The current study has adopted the European Burden of Disease Network consensus disease model for COVID-19 to estimate DALYs due to COVID-19. DALYs reflect the healthy life years lost due to diseases and constitute a morbidity component, i.e. years lived with disability (YLDs), and a mortality component, i.e. years of life lost (YLLs). Estimates of burden of disease indicators were calculated at the Belgian national level. Data inputs on the confirmed cases of COVID-19 and the COVID-19 deaths (both confirmed and suspected) covered the whole period of 2020 and 2021.

**Results**

In 2020, the total number of DALYs because of COVID-19 was estimated at 253,747 [253,108 – 254,479]. This is significantly higher compared to the DALYs in 2021, which were estimated at a total of 119,188 [118,548 – 119,921]. Around 98% of the DALYs in 2020 and 2021 are attributable to premature mortality because of COVID-19.

**Conclusions**

COVID-19 had a tremendous impact on the population health, specifically during the first year, comparable to the impact of cardiovascular disease in Belgium in 2020, and Alzheimer’s disease in 2021.

## A5 Direct impact of COVID-19 by estimating disability-adjusted life years at national level in France in 2020

### Romana Haneef^1^, Myriam Fayad^2^, Anne Fouillet^2^, Cécile Sommen^2^, Christophe Bonaldi^2^, Grant M A Wyper^3^, Sara Monteiro Pires^4^, Brecht Devleesschauwer^5, 6^, Antoine Rachas^7^, Panayotis Constantinou^7^, Daniel Levy-Bruhl^8^, Nathalie Beltzer^1^, Anne Gallay^1^

#### ^1^Department of Non-Communicable Diseases and Injuries, Santé Publique France, Saint-Maurice, France; ^2^Department of Data science, Santé Publique France, Saint-Maurice, France; ^3^School of Health & Wellbeing, University of Glasgow, Glasgow, United Kingdom; ^4^National Food Institute, Technical University of Denmark, Lyngby, Denmark; ^5^Department of Epidemiology and Public Health, Sciensano, Brussels, Belgium; ^6^Department of Translational Physiology, Infectiology and Public Health, Ghent University, Merelbeke, Belgium; ^7^Department of Strategy, Studies and Statistics, French National Health Insurance: Caisse nationale de l'assurance maladie (Cnam), Paris, France; ^8^Department of Infectious Diseases, Santé Publique France, Saint-Maurice, France

##### **Correspondence:** Romana Haneef (Romana.HANEEF@santepubliquefrance.fr)

*Archives of Public Health 2023*, **81(Suppl 2):**A5

Link to the full article: 10.1371/journal.pone.0280990

## A6 Burden of eating disorders in the European Region, 1990-2019

### Irena Ilic^1^ and Milena Ilic^2^

#### ^1^Faculty of Medicine, University of Belgrade, Belgrade, Serbia; ^2^Department of Epidemiology, Faculty of Medical Sciences, University of Kragujevac, Kragujevac, Serbia

##### **Correspondence:** Irena Ilic (ajrini10@gmail.com)

*Archives of Public Health 2023*, **81(Suppl 2):**A6

**Background**

Eating disorders (ED) are a public health issue due to the risk of increased mortality, disability, reduced quality of life, increased economic cost [1-3]. This study aimed to assess the burden of ED in the European Region.

**Methods**

A descriptive epidemiological study design was used. Data about ED were obtained from the Global Burden of Disease 2019 study. This study addresses the burden of ED by evaluating the disability-adjusted life-years (DALYs) [4]. The age-standardized rates (ASRs, expressed per 100,000) were presented. Joinpoint regression analysis was applied to calculate the average annual percent change (AAPC) with 95% confidence interval (CI) to evaluate trends in 1990-2019 [5].

**Results**

In both sexes together, the ASR of DALYs for ED in 2019 was the highest in Monaco (184.9 per 100,000), followed by Spain, Luxembourg and Austria (equally about 120.0), while the lowest rates were observed in Bosnia and Herzegovina, Republic of Moldova and Albania (equally about 20.0) (Figure 1).

Trend in ED DALYs significantly increased both in males (AAPC= +0.6%; 95%CI= 0.5 to 0.7) and females (AAPC= +0.7%; 95%CI= 0.6 to 0.7) in the European Region in 1990-2019 (Figure 2).

The highest rise of ED DALYs was observed in Western European countries (the Netherlands by +1.5% per year and Ireland by +1.3% per year), but an increasing trend was observed in almost all countries in the European Region, except Ukraine and Republic of Moldova, where stable trends were observed (Figure 3).

**Conclusions**

These results could help to better understand the burden of eating disorders which is crucial for improving their management.

**References**

1. GBD 2019 Diseases and Injuries Collaborators. Global burden of 369 diseases and injuries in 204 countries and territories, 1990-2019: a systematic analysis for the Global Burden of Disease Study 2019. Lancet. 2020; 396:1204-1222.
2. GBD 2019 Risk Factors Collaborators. Global burden of 87 risk factors in 204 countries and territories, 1990-2019: a systematic analysis for the Global Burden of Disease Study 2019. Lancet. 2020; 396:1223-1249.
3. Qian J, Wu Y, Liu F, et al. An update on the prevalence of eating disorders in the general population: a systematic review and meta-analysis. Eat Weight Disord. 2022; 27:415-428.
4. Global Burden of Disease Collaborative Network. Global Burden of Disease Study 2019 (GBD 2019) Results. Seattle, United States: Institute for Health Metrics and Evaluation (IHME), 2020. Available from http://ghdx.healthdata.org/gbd-results-tool (Accessed on: 29/08/2022)
5. Kim HJ, Fay MP, Feuer EJ, Midthune DN. Permutation tests for joinpoint regression with applications to cancer rates. Stat Med. 2000; 19:335-351.

**Fig. 1 (abstract A6):**
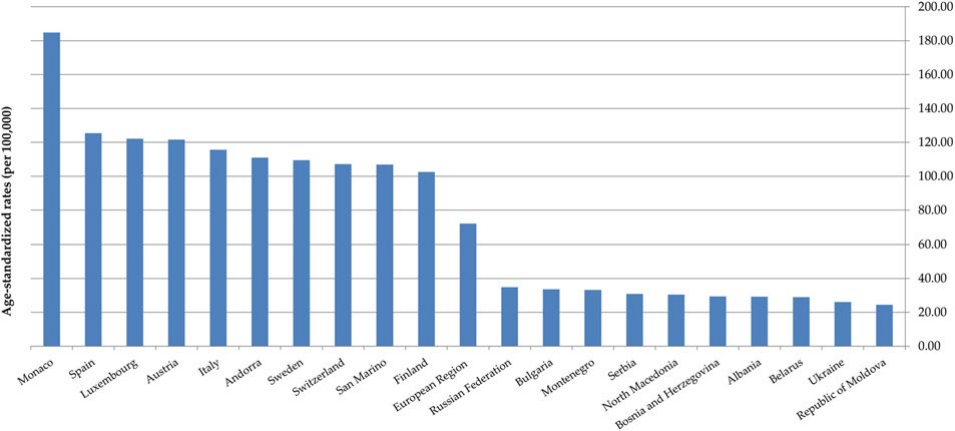
Eating disorders burden (DALYs) in both sexes together in the European Region, in 2019; 10 countries with the highest and the lowest rates.

**Fig. 2 (abstract A6):**
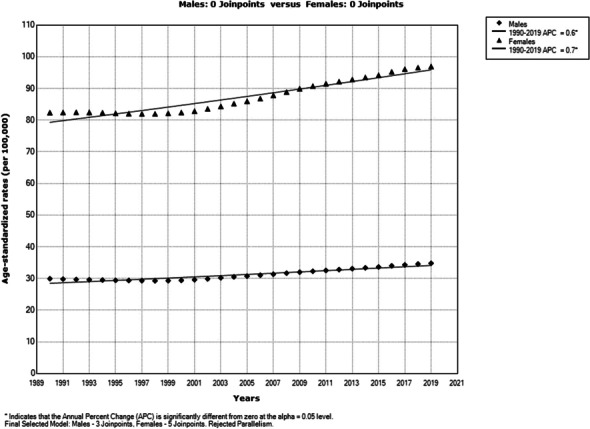
Trends of eating disorders burden (DALYs) in the European Region, by sexes, 1990-2019; a joinpoint regression analysis.

**Fig. 3 (abstract A6):**
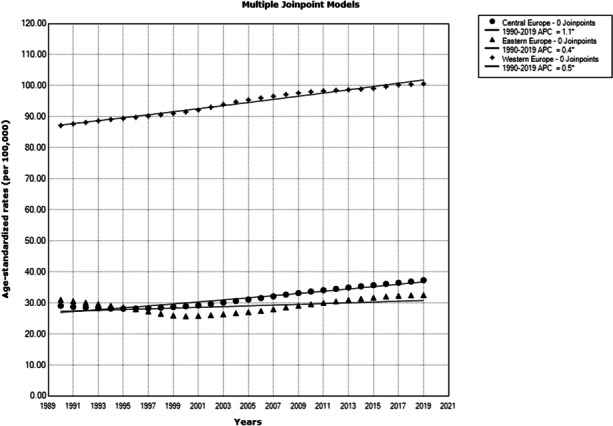
Trends of eating disorders burden (DALYs) in the European Region, 1990-2019, a joinpoint regression analysis: Central Europe (circle); Western Europe (diamond); Eastern Europe (triangle).

## A7 Prevalence estimation of musculoskeletal conditions in the Global Burden of Disease Study 2019: assessing risk of bias of primary data input studies and the certainty of GBD modelled estimates

### Javier Muñoz Laguna^1,2,3,4^, Milo A. Puhan^2^, Fernando Rodríguez Artalejo^1,5,6^, Robby De Pauw^7,8^, Grant M. A. Wyper^9,10^, Brecht Devleesschauwer^7,10^, João Vasco Santos^11,12,13^, Cesar A. Hincapié^2,3,4^

#### ^1^Department of Preventive Medicine, Public Health and Microbiology, School of Medicine, Universidad Autónoma de Madrid, Madrid, Spain; ^2^Epidemiology, Biostatistics and Prevention Institute, University of Zurich, Zurich, Switzerland; ^3^University Spine Centre Zurich (UWZH), Balgrist University Hospital, University of Zurich, Zurich, Switzerland; ^4^EBPI-UWZH Musculoskeletal Epidemiology Research Group, University of Zurich and Balgrist University Hospital, Zurich, Switzerland; ^5^CIBERESP (CIBER of Epidemiology and Public Health), Madrid, Spain; ^6^IMDEA Food Institute, CEI UAM and CSIC, Madrid, Spain; ^7^Department of Epidemiology and Public Health, Sciensano, Brussels, Belgium; ^8^Department of Rehabilitation Sciences, Ghent University, Ghent, Belgium; ^9^School of Health & Wellbeing, University of Glasgow, Glasgow, United Kingdom; ^10^Department of Translational Physiology, Infectiology and Public Health, Ghent University, Merelbeke, Belgium; ^11^MEDCIDS—Department of Community Medicine, Information and Health Decision Sciences, Faculty of Medicine, University of Porto, Porto, Portugal; ^12^CINTESIS—Centre for Health Technology and Services Research, Porto, Portugal; ^13^Public Health Unit, ACES Grande Porto V – Porto Ocidental, ARS Norte, Porto, Portugal

##### **Correspondence:** Cesar A. Hincapié (cesar.hincapie@uzh.ch)

*Archives of Public Health 2023*, **81(Suppl 2):**A7

**Background**

Global Burden of Disease (GBD) metrics are widely used within musculoskeletal epidemiology [1]. Yet, little is known about the primary data input studies that informed the GBD 2019 modelled prevalence estimates of low back pain (LBP), neck pain (NP), and knee osteoarthritis (knee OA) [2,3]. Furthermore, the certainty of GBD modelled prevalence estimates has not been explored in depth.

**Objective**

To describe and critically appraise the primary data input studies that underpinned the GBD 2019 modelled prevalence estimates of LBP, NP, and knee OA, in five countries (Australia, Brazil, Canada, Spain, and Switzerland), and to advance understanding of the certainty of GBD modelled prevalence estimates for these three musculoskeletal pain conditions.

**Methods**

Using the GBD 2019 Data Input Sources Tool, we identified all relevant primary data input studies, performed descriptive analyses, and assessed their risk of bias using a validated tool [4]. We proposed an approach to rate the certainty of the GBD 2019 modelled prevalence estimates of LBP, NP, and knee OA based on the Grading of Recommendations, Assessment, Development and Evaluation (GRADE) Guidelines 30—the GRADE approach to assessing the certainty of modelled evidence [5].

**Results**

There were 67 primary data input studies for LBP, 2 for NP, and 3 for knee OA for GBD epidemiological estimates between 1990 and 2019, for Australia (n=12 studies), Brazil (n=11), Canada (n=8), Spain (n=22), and Switzerland (n=19). Most studies were rated as moderate risk of bias due to concerns about study population representativeness, unclear case definitions with no verbal or diagram specification of anatomical pain location, and the use of assessment instruments with unknown psychometric properties and susceptible to misclassification. Based on GRADE criteria, the certainty of the GBD 2019 modelled prevalence estimates varied between very low and low mainly due to risk of bias and indirectness.

**Conclusions**

Beyond the limitations of primary data input studies for LBP, NP, and knee OA in GBD 2019, the certainty of GBD modelled prevalence estimates is limited. Future primary input studies with low risk of bias, and the optimal assessment of uncertainty in modelled outputs, will likely improve our confidence in GBD modelled estimates for musculoskeletal conditions.

**Acknowledgements**

The authors would like to acknowledge the networking support from COST Action CA18218 (European Burden of Disease Network), supported by COST (European Cooperation in Science and Technology).

**References**

1. Vos T, Lim SS, Abbafati C, Abbas KM, Abbasi M, Abbasifard M, et al. Global burden of 369 diseases and injuries in 204 countries and territories, 1990–2019: a systematic analysis for the Global Burden of Disease Study 2019. Lancet. 2020 Oct;396(10258):1204–22.
2. Tamrakar M, Kharel P, Traeger A, Maher C, O’Keeffe M, Ferreira G. Completeness and quality of low back pain prevalence data in the Global Burden of Disease Study 2017. BMJ Glob Health. 2021 May;6(5):e005847.
3. Maher C, Ferreira G. Time to reconsider what Global Burden of Disease studies really tell us about low back pain. Ann Rheum Dis. 2022 Mar 1;81(3):306–8.
4. Hoy D, Brooks P, Woolf A, Blyth F, March L, Bain C, et al. Assessing risk of bias in prevalence studies: modification of an existing tool and evidence of interrater agreement. J Clin Epidemiol. 2012 Sep 1;65(9):934–9.
5. Brozek JL, Canelo-Aybar C, Akl EA, Bowen JM, Bucher J, Chiu WA, et al. GRADE Guidelines 30: the GRADE approach to assessing the certainty of modeled evidence—An overview in the context of health decision-making. J Clin Epidemiol. 2021 Jan 1;129:138–50.

## A8 COVID-19 statistics in 47 European countries: How do relate the number of conducted tests, the number of cases and the number of deaths?

### Milena Santric Milicevic, Jovana Todorovic, Aleksandar Stevanovic, Zorica Terzic Supic, Vesna Bjegovic Mikanovic

#### Univeristy of Belgrade, Faculty of Medicine, Institute of Social Medicine, Belgrade, Serbia

##### **Correspondence:** Milena Santric Milicevic (milena.santric-milicevic@med.bg.ac.rs)

*Archives of Public Health 2023*, **81(Suppl 2):**A8

**Background**

Adequate testing for COVID-19 can prevent the spread of COVID-19, especially to populations at greatest risk of adverse outcomes, and help initiate timely and appropriate treatment of registered cases [1-5]. Especially effective was the combination of rapid testing with the preventive strategies [3], and the widespread testing have been proven to be effective in the avoidance of nationwide lockdowns [4]. The aim of this study was to examine the association between the cumulative number of tests conducted, cases of illness and death due to COVID-19, available for 47 European countries/territories.

**Methods**

From the Worldometer, a provider of global COVID-19 statistics for many countries around the world [6], the cumulative number of total registered COVID-19 cases, COVID-19 deaths and conducted COVID-19 tests, all calculated per 1,000,000 for 47 European countries/territories for the period from the beginning of COVID-19 outbreak until July 19th, 2022, were analyzed with the Pearson correlation coefficient using the SPSS v 26.

**Results**

There is a significant positive correlation (r=0.554, p<0.001) between the cumulative number of cases per 1,000,000 (on average 351223.3, range: 103,984-703,830) and tests performed per 1,000,000 (on average 4,337,325.13; range 451,098-21,918,199), while a significant negative correlation (r=-0.290, p<0.05) between the cumulative number of registered cases per 1,000,000 and the number of deaths per 1,000,000 (on average 2,437.02; range 128 - 5,453)

**Conclusion**

Our results indicate a significant positive association between a country's capacity to test cases of COVID-19, expressed as the cumulative number of tests, and to register cases of COVID-19, expressed as the cumulative number of reported cases. Future studies should reveal whether higher death rates from COVID-19 in countries with fewer reported cases are also associated with more unreported cases of COVID-19.

**References**

1. Mathieu E, Ritchie H, Rodés-Guirao L, Appel C, Giattino C, Hasell J, Macdonald B, Dattani S, Beltekian D, Ortiz-Ospina E, Roser M. Coronavirus Pandemic (COVID-19). 2020. Published online at OurWorldInData.org. Available from: 'https://ourworldindata.org/coronavirus' [Online Resource]
2. Watson J, Whiting PF, Brush JE. Interpreting a COVID-19 test results. BMJ. 2020: 12; 369: m1808. Doi: 10.1136/bmj.m1808.
3. Peeling RW, Heymann DL. Innovations in COVID-19 testing: the road from pandemic response to control. Lancet Infect Dis. 2021; 21: 1334-5
4. Abdin AF, Fang YP, Caunhye A, Alem D, Barros A, Zio E. An optimization model for planning testing and control strategies to limit the spread of a pandemic- The case of COVID-19. Eur J Oper Res. 2021
5. Sobczak M, Pawliczak R. COVID-19 mortality rate determinants in selected Eastern European countries. BMC Public Health. 2022; 22: 2088
6. Worldometerrs- Coronavirus Statistics- Serbia. 2022. Available from: https://www.worldometers.info/coronavirus/country/serbia/

## A9 Disability-Adjusted Life Years for Occupational Cancer Diseases from 2010–2020: Data from regional Register of Occupational Diseases in Federation Bosnia and Herzegovina

### Nurka Pranjic^1,2^

#### ^1^Department of Occupational Medicine, School of Medicine, University of Tuzla, 75000 Tuzla, Bosnia and Herzegovina; ^2^Clinic of Occupational Pathology and Toxicology, University Institute of Primary Health, 75000 Tuzla, Bosnia and Herzegovina

##### **Correspondence:** Nurka Pranjic (pranicnurka@hotmail.com)

*Archives of Public Health 2023*, **81(Suppl 2):**A9

**Background**

Occupational cancers suffer workers who are long-term exposed to occupational carcinogen factors in the working environment after a long latent period. This study aims to estimate the regional burden of occupational cancer (OC) in the Federation of Bosnia and Herzegovina from 2010–2020 using the disability-adjusted life years data (DALYs) as a health measure. Data from 50 OCs out of 135 (37%) of all verified occupational diseases (ODs) from the regional Register of occupational diseases (ODs).

**Methods**

The years of life lost (YLL) were estimated with the remaining years lost of standardized expected years of life and mortality number incidence. The number of years lived with a disability and spent time in states of reduced health (YLD) do the multiplication of the number of incidence cases, duration, and disability weight. Disability-adjusted life years mean the years lived with disability (DALYs) were estimated using incidence rate per 100 000. The DALYs of OCs calculated as the arithmetic addition of YLL and YLD.

**Results**

In the study period, 26 deaths from all occupational cancers founded, and 23 cancer-attributable deaths were in male workers (88%). The results shed light on the main six regional carcinogenic risks: coke-oven emissions (58%), ionizing radiations (x-rays) in the health care sector (24%), benzene (10%), asbestos (4%), vinyl chloride (2%), and viruses as biological carcinogens (2%), see Table 1.

The prevalent were larynx carcinoma (34%), lung cancer (22%), and Leucemia (18%). The most frequent cancers as outcomes among female workers were breast, kidney, ovarian, and vesicae urinary. For all malignant tumors, DALY was at 7983,094, and the highest DALY for cancer of the larynx, 3768,464, and lung cancer, 3411,536.

**Conclusion**

The present study is the first research in Bosnia and Herzegovina that investigated the DALYs of professional carcinomas and the outcome of exposure to workplace carcinogens. Occupational cancers have a high frequency compared to all other ODs in the ten-year research period. Coke oven plant workers have an occupational cancer epidemic, and they are not exposed to just one carcinogen but to a combination of carcinogens. The incidence of x-ray (associated) cancers in healthcare workers are worrying. Study results provide the need to expand cancer prevention at the workplace, stop exposure to coke oven emissions, x-ionizing radiation, benzene, or its homologs without continued preventive activities, and provide cancer screening and awareness programs.

**Table 1 (abstract A9):** Demographics and data of working history

	No	Mean	SD*
Duration of occupational exposure to carcinogen/s (years)	50	21,960	8,708
Length of service (years)	50	26,040	8,845
Age	50	50,880	7,808
Duration of exposure to occupational carcinogens	50	21,960	8,708
Gender, male	40/50		
Workability loss	48/50		
Death	26/50		
Exposure to occupational carcinogens			
3.1.12.† Coke oven emissions	29/50		
3.1.10. Ionizing radiations (x-rays), health care sector	12/50		
3.1.9. Toxic nitro- and amino-derivatives of benzene	5/50		
3.1.1. Asbestos	2/50		
3.1.7. Vinyl chloride	1/50		
3.1.21. Viruses (healthcare children)	1/50		

*standard deviation; † ILO- classification of occupational carcinogens

**Table 2 (abstract A9):** Disability-Adjusted Life Years for Occupational Cancer Diseases from 2010–2020

Occupational cancer	No	Incidence rate	DW	Cancer years	YLL	YLD	DALY
Esophageal cancer	4	8000	2,904	5	1970,3	11,616	1981.916
Stomach cancer	4	8000	1,176	8	1730,5	37,632	1768,139
Liver carcinoma	2	4000	0,588	4	880,8	4,704	888,504
Cancer of larynx	9	18000	3,388	17	3250,1	518,364	3768,464
Lung cancer	8	16000	3,872	11	3070,8	340,736	3411,536
Melanoma	4	8000	1,176	3	1630,8	14,112	1644,912
Breast carcinoma	3	6000	0,882	3	1280,4	7,938	1288,338
Ovary carcinoma	1	2000	0,484	2	540,3	0,968	541,268
Prostate carcinoma	1	2000	0,294	3	300,3	0,882	301,182
Bladder carcinoma	1	2000	0,484	2	300,3	0,968	301,968
Brain neoplasm	2	4000	1,936	2	930,7	7,744	938,444
Leucemia	4	8000	1,176	9	1870,6	42,336	1912,936
Kidney carcinoma	3	6000	1,452	7	1430,2	30,492	1460,692
Other cancers	4	8000	0,588	6	1880,1	14,112	1894,212
All sites	50	100000	20,4	82	6950,5	1032,594	7983,094

## A10 Results from GBD 2019 in Montenegro

### Lidija Scepanovic^1^, Natasa Terzic^2^

#### ^1^Center for Health Policy and Management, Institute of Public Health of Montenegro, Podgorica, Montenegro; ^2^Center for Data Records and Research in Public Health, Institute of Public Health of Montenegro, Podgorica, Montenegro

##### **Correspondence:** Lidija Scepanovic (lidija.scepanovic@ijzcg.me)

*Archives of Public Health 2023*, **81(Suppl 2):**A10

**Background**

The Global burden of disease (GBD) represents a powerful source of information for population health worldwide, contributing to better understand the true nature of the country’s health challenges [1]. We summarized the data of the health status for Montenegro in 2019 followed by the changes from 2009.

**Methods**

GBD 2019 estimated disease burden due to 369 diseases and injuries, and 86 risk factors and 54 new risk-outcomes [1,2]. Mortality and DALYs, risk factors, and progress towards the Universal Health Coverage are presented.

**Results**

There were 6,793 deaths in Montenegro in 2019, with 64.34% of them aged 70+ years. Noncommunicable diseases were the main cause of death (94.02%), followed by Injuries (4.55%), and Communicable diseases (1.43%). The leading causes of death were: Stroke (27.72%), Ischemic heart disease (21.91%), Lung cancer (7.81%), Cardiomyopathy (3.83%) and Alzheimer's (2.72%). Greater increases in mortality were observed in Alzheimer's, other cancers, diabetes, other Cardio vascular diseases compared to 2009. Stroke, Ischemic heart disease and Lung cancer remain the top causes of DALYs in 2019, while Diabetes moved to 4th position. Tobacco is the most relevant risk factor, followed by High blood pressure and increase in High body-mass index and High fasting plasma glucose. The Universal Health Coverage index was improved reaching 66 points in 2019, with average change per year of 0.4% since 2010 [3].

**Conclusion**

Stroke, Ischemic heart disease and Lung cancer remain the top causes of death and disability in 2019 in Montenegro, followed by diabetes. Tobacco is still the main health issue to be addressed.

**References**

1. GBD 2019 Diseases and Injuries Collaborators. Global burden of 369 diseases and injuries in 204 countries and territories, 1990–2019: a systematic analysis for the Global Burden of Disease Study 2019. The Lancet. 2020;396(10258):1204–22.
2. GBD Results Tool | GHDx. Ghdx.healthdata.org. 2021. http://ghdx.healthdata.org/gbd-results-tool.
3. Measuring universal health coverage based on an index of effective coverage of health services in 204 countries and territories, 1990–2019: a systematic analysis for the Global Burden of Disease Study 2019. The Lancet. 2020; DOI: 10.1016/S0140-6736(20)30750-9.

## A11 Environmental burden of disease estimates and their use for scientific policy consulting

### Myriam Tobollik

#### Section Environmental Medicine and Health Effects Assessment, German Environment Agency, Berlin, Germany

##### **Correspondence:** Myriam Tobollik (myriam.tobollik@uba.de)

*Archives of Public Health 2023*, **81(Suppl 2):**A11

**Background**

In Germany, scientific policy consulting is an integral part of the political system. One public health method developed with the aim of providing scientific policy advice is the environmental burden of disease method. The theoretical and scientific applications are diverse, but the knowledge gained with this method is hardly used in scientific policy advice.

**Methods**

As part of my doctorate studies, I examined the opportunities and limitations of the environmental burden of disease method for scientific policy advice from a scientific point of view. Four application examples and a review article were assessed. In addition, two communication examples were developed.

**Results**

The comparability of environmental burden of disease estimates is one of the advantages of the method, although this could only be proven to a limited extent on the basis of the researched examples. The differences in the input data and its availability, the level of detail and the geographical area hindered the comparability. For a political uptake it is necessary to describe the differences and the reasons for them. With regard to the communication of the complex calculations, it is necessary to create a basic understanding of the metrics through various communication media among decision makers.

**Conclusion**

Besides several limitations, such as comparability and intelligibility, environmental burden of disease is a valid method for scientific policy advising. It can be used to give an order of magnitude of the impact of environmental risk factors on health.

## A12 Methodological challenges to assess the environmental burden of disease for children in Germany – Findings of the UKAGEP-project

### Myriam Tobollik^1^, Sarah Kienzler^2^, Dietrich Plass^2^, Dirk Wintermeyer^2^

#### ^1^Section Environmental Medicine and Health Effects Assessment, German Environment Agency, Berlin, Germany; ^2^Section Exposure Assessment and Environmental Health Indicators, German Environment Agency, Berlin, Germany

##### **Correspondence:** Myriam Tobollik (myriam.tobollik@uba.de)

*Archives of Public Health 2023*, **81(Suppl 2):**A12

**Background**

Generally, children are a relatively healthy population. However, exposure to risk factors during childhood can lead to adverse health effects later in life. One main aim of the UKAGEP-project was to estimate the environmental burden of disease attributable to a selected set of environmental risk factors for children aged 3 to 17 years in Germany.

**Methods**

We used the environmental burden of disease-method and its core measure, the disability-adjusted life year. Where possible, current exposure and health data for children were derived from the population-representative German Environmental Survey (GerES V 2014-2017). For selected risk factors systematic literature reviews were performed to identify exposure-response functions.

**Results**

Environmental burden of disease quantifications could be performed for only four out of 18 risk factors: secondhand smoke, Bisphenol A, traffic noise, and particulate matter. This is due to several limitations encountered during the estimation process. One major issue was that we could hardly use the survey data for the assessments, because for several risk factors, the existing exposure levels were too low and thus would not result in any disease burden. Further, it was challenging to combine exposure-response functions identified by the reviews with the survey data. Therefore, other exposure sources and health data were used, which, however, did not only focus on children but also on adults (traffic noise and particulate matter).

**Conclusions**

The research project showed that the application of the environmental burden of disease approach differed between the four risk factors consequently hampering the direct comparisons of the results.

**Acknowledgement**

This project was funded by the German Federal Ministry of Education and Research under the grant number 01KX1406.

## A13 Inequalities in the burden of disease of 44 European countries from 1990 to 2019

### Orsolya Varga^1^, Jonila Gabrani^2^, Periklis Charalampous^3^, Grant MA Wyper^4^, Sarah Cuschieri^5^, Diana Alecsandra Grad^6^, Brigid Unim^7^, Enkeleint A Mechili ^8,9^, José Chen-Xu^10,11^, Elena von der Lippe^12^, Juanita A Haagsma^3^

#### ^1^Department of Public Health and Epidemiology of Faculty of Medicine, University of Debrecen, Debrecen Hungary; ^2^University of Basel, Faculty of Medicine, Basel, Switzerland; ^3^Department of Public Health, Erasmus MC University Medical Center, Rotterdam, The Netherlands; ^4^Place and Wellbeing Directorate, Public Health Scotland, UK; ^5^Faculty of Medicine and Surgery, University of Malta, Msida, Malta; ^6^Department of Public Health, Babeş-Bolyai University, Cluj-Napoca, Romania; ^7^Department of cardiovascular, endocrine-metabolic diseases and aging, Istituto Superiore di Sanità, Rome, Italy; ^8^Department of Healthcare, Faculty of Health, University of Vlora, Albania; ^9^Clinic of Social and Family Medicine, School of Medicine, Crete, Greece; ^10^Public Health Unit, Primary Healthcare Cluster Baixo Mondego, Coimbra, Portugal; ^11^National School of Public Health, NOVA University of Lisbon, Lisbon, Portugal; ^12^Department of Epidemiology and Health Monitoring, Robert Koch Institute, Berlin, Germany

##### **Correspondence:** Orsolya Varga (varga.orsolya@med.unideb.hu)

*Archives of Public Health 2023*, **81(Suppl 2):**A13

**Background**

Health inequalities are unjust and avoidable disparities in health status between countries or sub-groups of a population. Comparing Disability Adjusted Life Years (DALYs) rates across populations can facilitate the understanding of health inequalities, serving as a basis for evidence based policy interventions [1]. Insights into cause-specific inequalities across countries and over time, using the DALY metric are currently limited in Europe. The objective of this study was to assess inequalities in DALY rates between 44 countries in Europe over time, by all-cause and cause-specific category.

**Methods**

We performed a descriptive study using the Global Burden of Disease 2019 results on age-standardized DALY in 44 European countries from 1990 to 2019 [2]. Inequality between these countries was reported using the ratio of DALY rate for the highest-ranking country to the lowest-ranking country in each year expressing the difference between DALY experiences in Europe.

**Results**

From 1990 to 2019, the all-cause DALY rate ratio fluctuated between 1.8 in 1990 (highest ranking country: Republic of Moldova; lowest ranking country: San Marino), peaked to 2.4 in 1994 and 2005 (highest ranking country: Russian Federation; lowest ranking country: San Marino) and 2.0 in 2019 (highest ranking country: Ukraine; lowest ranking country: Iceland). In 2019, high variation in DALY rates was observed for most diseases, especially for cardiovascular diseases (6.9 times higher in Ukraine compared to France) and HIV&AIDS and sexually transmitted infections (66 times higher in Ukraine compared to Finland).

**Conclusions**

Since health inequalities are mainly rooted in economic and social causes, they require a comprehensive solution. Still, the health sector's potential, especially prevention efforts targeting non-communicable diseases, should not be overlooked.

**References**

1. GBD 2019 Diseases and Injuries Collaborators Global burden of 369 diseases and injuries in 204 countries and territories, 1990–2019: a systematic analysis for the Global Burden of Disease Study 2019. Lancet. 2020;396:1204–1222.
2. Global Burden of Disease Collaborative Network. Global Burden of Disease Study 2019 (GBD 2019) Reference Life Table. Seattle, United States of America: Institute for Health Metrics and Evaluation (IHME), 2021.

## A14 The changing pattern of trends in liver cancer burden in the European Region

### Irena Ilic^1^ and Milena Ilic^2^

#### ^1^Faculty of Medicine, University of Belgrade, Belgrade, Serbia; ^2^Department of Epidemiology, Faculty of Medical Sciences, University of Kragujevac, Kragujevac, Serbia

##### **Correspondence:** Irena Ilic (ajrini10@gmail.com)

*Archives of Public Health 2023*, **81(Suppl 2):**A14

**Background**

Liver cancer (LC) remains one of the major public health issues worldwide [1-3]. This study aimed to reveal the burden of LC attributable to five specific risk factors in the European Region in 1990-2019.

**Methods**

An ecological trend study was conducted. This study used the Global Burden of Disease Study 2019 data (including disability-adjusted life years - DALYs of LC, as well as LC due to alcohol use, hepatitis B, hepatitis C, nonalcoholic steatohepatitis, other causes) [4]. The DALYs are presented as age-standardized rates (ASRs) per 100,000 population. The trends in the LC burden were assessed using joinpoint regression analysis, by the average annual percent change (AAPC) with 95% confidence interval (CI) [5].

**Results**

In both sexes together, the highest ASR of DALYs for LC in the European Region was attributed to alcohol use – 32.1 per 100,000 in 2019, followed by hepatitis C (28.2) and hepatitis B (19.2), and then nonalcoholic steatohepatitis and other causes (equally about 6.0) (Figure 1).

The DALYs for LC attributable to hepatitis C are concentrated in Western European countries (ASR=36.0), with a significantly increasing trend in the last three decades (AAPC=+0.5; 95%CI=0.3 to 0.6) (Figure 2).

The rising trends in ASRs of DALYs for LC in Eastern Europe are particularly worrying, both for LC due to alcohol use (AAPC=+2.9; 95%CI=2.6 to 3.3) and hepatitis C (AAPC=+2.4; 95% CI=2.2 to 2.7) and hepatitis B (AAPC=+2.2; 95%CI=1.8 to 2.5).

**Conclusions**

These epidemiological data indicate that additional preventive strategies for LC are needed to further reduce disease burden in the European Region.

**References**

1. GBD 2019 Diseases and Injuries Collaborators. Global burden of 369 diseases and injuries in 204 countries and territories, 1990-2019: a systematic analysis for the Global Burden of Disease Study 2019. Lancet. 2020; 396:1204-1222.
2. GBD 2019 Risk Factors Collaborators. Global burden of 87 risk factors in 204 countries and territories, 1990-2019: a systematic analysis for the Global Burden of Disease Study 2019. Lancet. 2020; 396:1223-1249.
3. Mårdh O, Quinten C, Amato-Gauci AJ, Duffell E. Mortality from liver diseases attributable to hepatitis B and C in the EU/EEA - descriptive analysis and estimation of 2015 baseline. Infect Dis (Lond). 2020: 52:625-637.
4. Global Burden of Disease Collaborative Network. Global Burden of Disease Study 2019 (GBD 2019) Results. Seattle, United States: Institute for Health Metrics and Evaluation (IHME), 2020. Available from http://ghdx.healthdata.org/gbd-results-tool (Accessed on: 29/08/2022)
5. Kim HJ, Fay MP, Feuer EJ, Midthune DN. Permutation tests for joinpoint regression with applications to cancer rates. Stat Med. 2000; 19:335-351.

**Fig. 1 (abstract A14):**
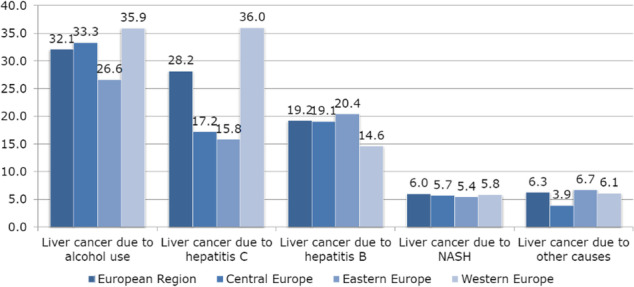
The burden (DALYs, ASRs per 100,000) of liver cancer in both sexes together in European Region, by selected risk factors, in 2019.

**Fig. 2 (abstract A14):**
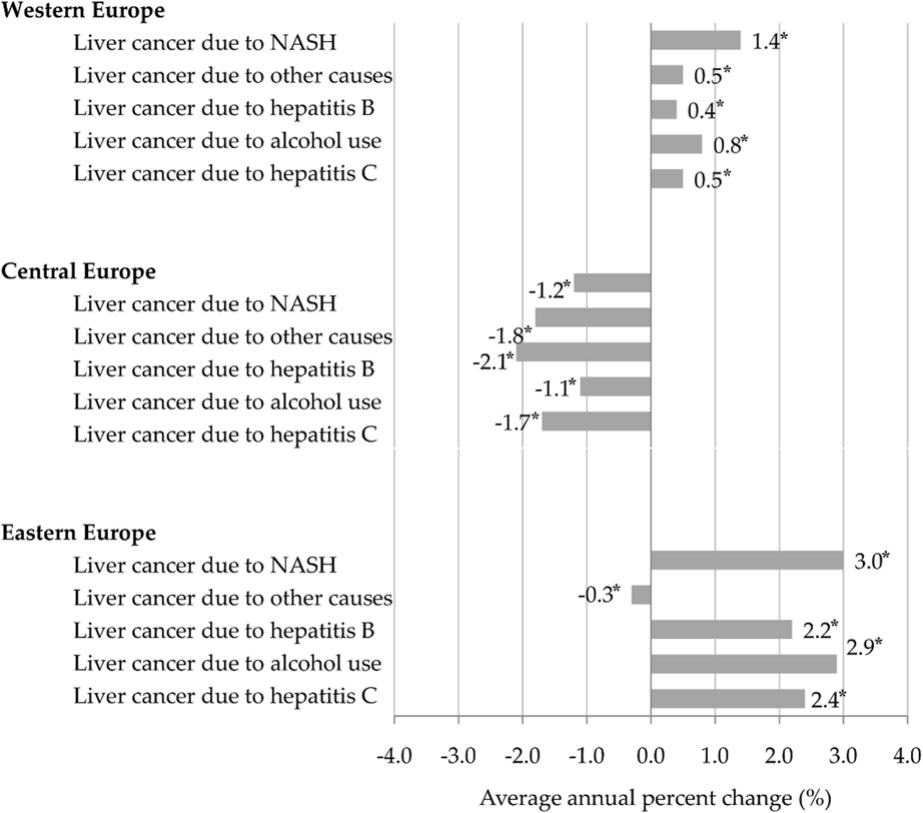
Trends of liver cancer burden (DALYs, ASRs per 100,000) attributed to selected risk factors, in both sexes together in the European Region, 1990-2019; a joinpoint regression analysis. (* p<0.05).

## A15 The impact of high fasting plasma glucose on the burden of lung cancer in the European Region

### Irena Ilic^1^ and Milena Ilic^2^

#### ^1^Faculty of Medicine, University of Belgrade, Belgrade, Serbia; ^2^Department of Epidemiology, Faculty of Medical Sciences, University of Kragujevac, Kragujevac, Serbia

##### **Correspondence:** Irena Ilic (ajrini10@gmail.com)

*Archives of Public Health 2023*, **81(Suppl 2):**A15

**Background**

Lung cancer (LC) is the leading cause of cancer-related deaths worldwide [1,2]. The role of high fasting plasma glucose (HFPG) in LC burden is still unclear [2,3]. This study aimed to estimate disability pattern for LC attributed to HFPG in European Region.

**Methods**

A descriptive epidemiological study was done. Data were obtained from the database of the Global Burden of Disease 2019 study [4]. The disability-adjusted life-years (DALYs) for HFPG as a risk factor for LC were presented. The age-standardized rates (ASRs) of DALYs were expressed per 100 000 population. Trends in LC attributable to HFPG in 1990-2019 were estimated using joinpoint regression analysis [5].

**Results**

The percentage of DALYs of LC attributable to HFPG was 8.6% in males (accounting for 641 718 DALYs) and 8.0% in females (accounting for 227 070 DALYs) in 2019 in the European Region (Figure 1).

In 2019, Central Europe was the worst-affected subregion, with ASR of DALYs due to LC attributable to HFPG of 146.6 per 100 000 in males and 37.6 per 100 000 in females (Figure 2).

The ASRs DALYs increased significantly in both sexes in Central Europe (by +0.8% per year in males and by 3.4% per year in females) from 1990 through 2019 (Figure 3). In contrast, the ASRs of DALYs decreased significantly in males only in Western Europe (by -0.4% per year) and Eastern Europe (by -1.2% per year).

**Conclusions**

Our study noted sex differences in lung cancer burden due to high fasting plasma glucose, that need be evaluated in further analytical research.

**References**

1. GBD 2019 Diseases and Injuries Collaborators. Global burden of 369 diseases and injuries in 204 countries and territories, 1990-2019: a systematic analysis for the Global Burden of Disease Study 2019. Lancet. 2020; 396:1204-1222.
2. GBD 2019 Risk Factors Collaborators. Global burden of 87 risk factors in 204 countries and territories, 1990-2019: a systematic analysis for the Global Burden of Disease Study 2019. Lancet. 2020; 396:1223-1249.
3. Argirion I, Weinstein SJ, Mannisto S, Albanes D, Mondul AM. Serum insulin, glucose, indices of insulin resistance, and risk of lung cancer. Cancer Epidemiol Biomark Prev. 2017; 26:1519–1524.
4. Global Burden of Disease Collaborative Network. Global Burden of Disease Study 2019 (GBD 2019) Results. Seattle, United States: Institute for Health Metrics and Evaluation (IHME), 2020. Available from http://ghdx.healthdata.org/gbd-results-tool (Accessed on: 29/08/2022)
5. Kim HJ, Fay MP, Feuer E. J, Midthune DN. Permutation tests for joinpoint regression with applications to cancer rates. Stat Med. 2000; 19:335-351.

**Fig. 1 (abstract A15):**
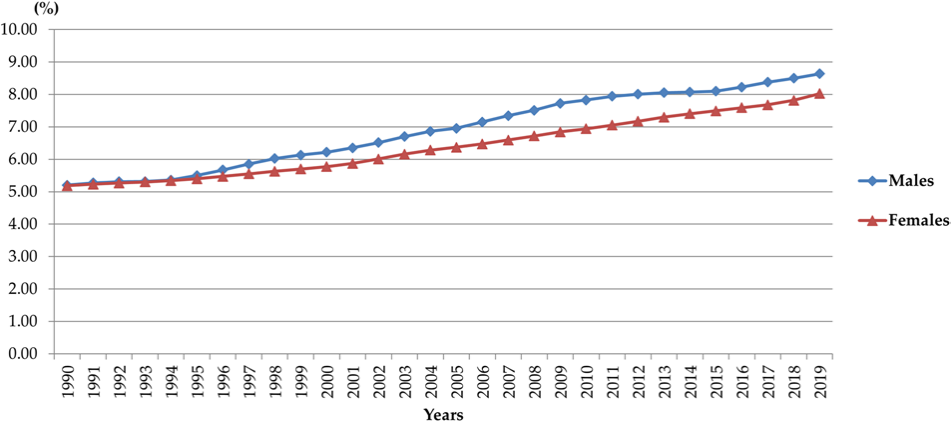
The contribution (%) of high fasting plasma glucose to lung cancer burden (DALYs) in the European Region, by sex, 1990-2019.

**Fig. 2 (abstract A15):**
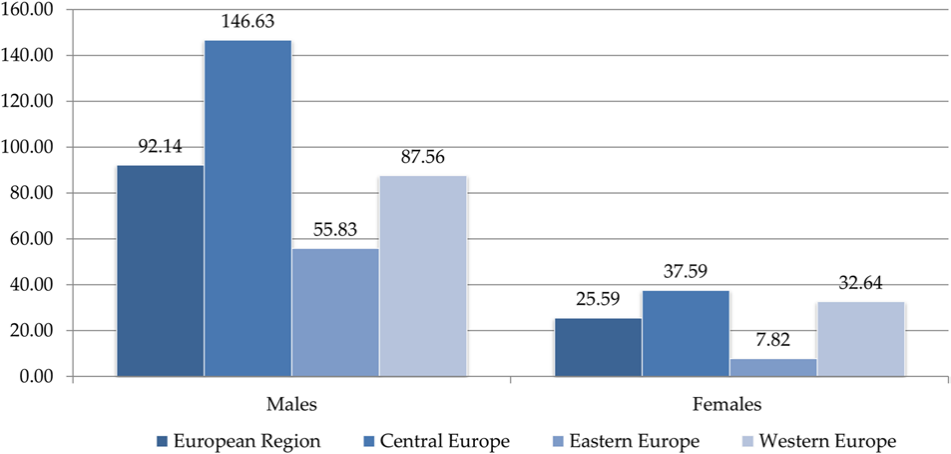
The burden (DALYs, ASRs per 100,000) of lung cancer attributed to high fasting plasma glucose in European Region, by sex, in 2019.

**Fig. 3 (abstract A15):**
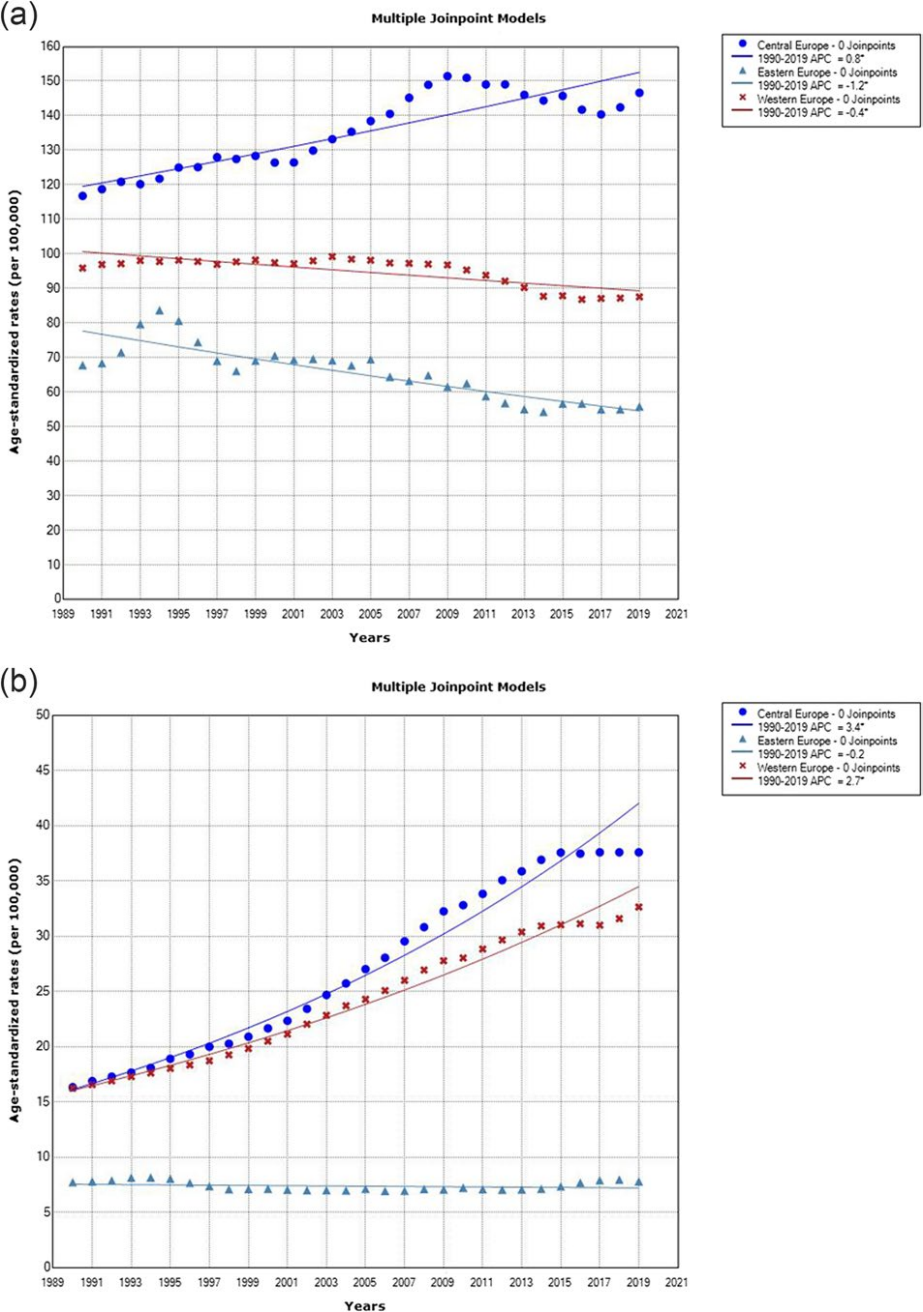
Lung cancer burden (DALYs) attributed to high fasting plasma glucose in the European Region, 1990-2019, a joinpoint regression analysis: (a) Males; (b) Females. Central Europe (circle); Western Europe (cross); Eastern Europe (triangle).

## A16 Use of DALYs in Risk Benefit Assessment modeling to evaluate edible insects as red meat replacers (the NovRBA Project)

### Ermolaos Ververis^1,2^, Aikaterini Niforou^1^, Géraldine Boué^3,4^, Sara Monteiro Pires^5^, Morten Poulsen^5^, Lea S. Jakobsen^5^, Androniki Naska^1^

#### ^1^Dept. of Hygiene, Epidemiology and Medical Statistics, School of Medicine, National and Kapodistrian University of Athens (NKUA), Athens, Greece; ^2^European Food Safety Authority (EFSA), Parma, Italy; ^3^French National Institute for Agricultural Research (INRAE), Paris, France; ^4^Nantes-Atlantic National College of Veterinary Medicine, Food Science and Engineering (ONIRIS), Nantes, France; ^5^Division of Diet, Disease Prevention and Toxicology, National Food Institute, Technical University of Denmark (DTU), Kongens Lyngby, Denmark

##### **Correspondence:** Ermolaos Ververis (ermolaos.ververis@efsa.europa.eu)

*Archives of Public Health 2023*, **81(Suppl 2):**A16

**Background**

The NovRBA project (Novel foods as red meat replacers-an insight using Risk-Benefit Assessment methods) investigated the health impact of replacing red meat with an edible insect form (cricket powder) on the Danish, French and Greek healthy adult populations^1^.

**Methods**

Food components of red meat and cricket flour linked to health outcomes were identified^2^, prioritized, and selected^3^. Epidemiological studies were used to identify causal associations between exposure to chemical hazards/dietary components and health outcomes, and the respective burden of disease was quantified through the estimation of DALYs per case, based on DALYs retrieved from the Global Burden of Disease (GBD) database^4^ and incidence rates from WHO databases^5^. When available, country-specific DALYs were used. Concentration and prevalence of microbiological hazards were estimated for the insect-based product, whereas the cases attributed to meat consumption were estimated based on disease burden and source attribution. Estimates for diet-disease associations were obtained from dose-response meta-analyses.

**Results**

The overall health impact was estimated in DALYs. Substitution could save around 6,572 DALYs (per 100,000 population) in Denmark, 21,972 DALYs (per 100,000 population) in France and 8,753 DALYs (per 100,000 population) in Greece, mainly due to the overall beneficial nutritional and microbiological impacts.

**Conclusion**

The NovRBA project developed and tested a harmonized RBA approach for dietary substitution. Each country had different DALY results due to differences in food intake and their current incidence of diseases. The absence of DALY values for specific health outcomes and the need of utilizing disability weights to estimate these values were among the knowledge gaps identified.

Disclaimer: EV is employed with EFSA in the Nutrition and Food Innovation Unit that provides scientific and administrative support to the Panel on Nutrition, Novel Foods and Food Allergens. The NovRBA project was supported by EFSA [Grant number GP/EFSA/GP/EFSA/ENCO/2018/03-GA01] and was coordinated by NKUA. However, the present work is published under the sole responsibility of the authors and may not be considered an EFSA scientific output. The positions and opinions presented in this work are those of the authors alone and do not necessarily represent the views or scientific work of EFSA.

**References**

1. Naska A, Ververis E, Niforou A, Pires SM, Poulsen M, Jakobsen LS, Becker N, Lohmann M, Tesson V, Federighi M, Boué G. Novel foods as red meat replacers–an insight using Risk Benefit Assessment methods (the NovRBA project). EFSA Supporting Publications. 2022 May;19(5):7316E.
2. Ververis E, Boué G, Poulsen M, Pires SM, Niforou A, Thomsen ST, Tesson V, Federighi M, Naska A. A systematic review of the nutrient composition, microbiological and toxicological profile of Acheta domesticus (house cricket). Journal of Food Composition and Analysis. 2022 Aug 27:104859.
3. Boué G, Ververis E, Niforou A, Federighi M, Pires SM, Poulsen M, Thomsen ST, Naska A. Risk-Benefit Assessment of foods: development of a methodological framework for the harmonized selection of nutritional, microbiological and toxicological components. Front Nutr.:2528.
4. Global Burden of Disease Study 2019, http://www.healthdata.org/gbd/2019
5. World Health Organization (WHO), European Health for All database (HFA-DB), https://gateway.euro.who.int/en/datasets/european-health-for-all-database/

## A17 Burden of rehabilitation in Serbia

### Branko Vujkovic^1^, Branka Markovic^2^, Igor Dragicevic^1^

#### ^1^Public Health Institute, Sabac, Serbia; ^2^Faculty of Sport and Physical Education, University of Belgrade, Belgrade, Serbia

##### **Correspondence:** Branko Vujkovic (vujkovicb@gmail.com)

*Archives of Public Health 2023*, **81(Suppl 2):**A17

**Background**

Rehabilitation has often been construed to be as a very specialized and expensive service for the few [1]. By comparing two studies on the burden of diseases in the world in 1990. and 2010. [1,2], it was shown that pain in the lumbar spine rose from eleventh to sixth place of diseases in terms of disability-adjusted life years (DALY). Considering the increasing age of the population, risk factors, increased incidence of musculoskeletal disorders in general, back pain with depression is becoming a leading cause of disability [3].

**Methods**

WHO, together with Institute for Health Metrics and Evaluation (IHME), has developed a Rehabilitation Need Estimator (RNE); a web-based tool that provides data visualizations of the estimated need for rehabilitation globally [2,4]. In our work we analysed key findings based on RNE, and the need for rehabilitation in Serbia.

**Results**

Global key findings showed that 310 million years have been lived with disability. There has been a 69.4% increase in years lived with disability between 1990 and 2019. Serbia key findings in 2019 showed that approximately 7 in 15 people could benefit from rehabilitation; 4.1 million people experienced conditions that could benefit from rehabilitation; 1.2 million people have been affected by fractures; 490 thousand years have been lived with disability. There has been a 7.8% increase in years lived with disability between 1990 and 2019.

**Conclusions**

Rehabilitation should be integrated as an essential strategy in long-term care, as its main goal is to improve limitations in everyday functioning due to aging or underlying health conditions. Optimizing functioning is the ultimate objective of rehabilitation, regardless of who the beneficiary is, who delivers it, or the context in which rehabilitation is delivered. The traditional workforce in primary care settings need to be trained in assessing rehabilitation needs and in the delivery of rehabilitation interventions that address common health problems.

**References**

1. Cieza A, Causey K, Kamenov K, Hanson SW, Chatterji S, Vos T. Global estimates of the need for rehabilitation based on the Global Burden of Disease study 2019: a systematic analysis for the Global Burden of Disease Study 2019. Lancet. 2021 Dec 19;396(10267):2006-2017. doi: 10.1016/S0140-6736(20)32340-0. Epub 2020 Dec 1. Erratum in: Lancet. 2020 Dec 4;: PMID: 33275908; PMCID: PMC7811204.
2. Global estimates of the need for rehabilitation. Available from: [https://www.who.int/teams/noncommunicable-diseases/sensory-functions-disability-and-rehabilitation/global-estimates-of-the-need-for-rehabilitation]
3. Murray CJ, Lopez AD, Jamison DT. The global burden of disease in 1990: summary results, sensitivity analysis and future directions. Bull World Health Organ. 1994;72(3):495-509. PMID: 8062404; PMCID: PMC2486716.
4. WHO Rehabiltation Need Estimator. Available from: [https://vizhub.healthdata.org/gbd-compare/]

## A18 The burden of disease in Germany and its regions - disability-adjusted life years (DALY) from the BURDEN 2020 study

### Annelene Wengler, Michael Porst, Janko Leddin, Aline Anton, Caoimhe Cawley, Elena von der Lippe, Alexander Rommel

#### Departement 2 – Epidemiology and Health Monitoring, Robert Koch Institute, Berlin, Germany

##### **Correspondence:** Annelene Wengler (wenglera@rki.de)

*Archives of Public Health 2023*, **81(Suppl 2):**A18

**Background**

Within the BURDEN 2020 project carried out by Robert Koch Institute, the German Environmental Agency, and the Scientific Institute of AOK burden of disease (BoD) estimates for Germany at the national and regional level become available for the first time. By focusing on Disability Adjusted Life Years (DALY) and comparing age-groups and regions we gain insight into the population health patterns in Germany.

**Methods**

DALY are quantified as the deviation of the population’s health from an "ideal" health status in the unit of life years. They are calculated as the sum of *years of life lost due to death* (YLL) and *years of life lost due to disability* (YLD). Calculations are based on different primary and secondary data sources, especially causes of death statistics, epidemiological survey data, and statutory health insurance data.

**Results**

For DALY-estimation 19 important diseases/injuries were considered. *Ischemic heart disease* contributes the most to the overall BoD, followed by *lower back pain* and *lung cancer*. In women, *headache disorders* and *dementias* account for more DALY as compared to men. Men have a higher BoD from *lung cancer* and *alcohol use disorders*. *Pain disorders* and *alcohol use disorders* are most relevant in younger adulthood. The burden due to cardiovascular disease, COPD, and diabetes mellitus increases with age, and also varies by region. All results are available through the website https://www.daly.rki.de (see also 1, 2).

**Conclusion**

BoD estimates main aim is the inter-cause comparison by age, sex, and region. Accordingly, the DALY-results enable a broader insight into population health, better public health reporting and hence support health-policy decision making and its evaluation.

Acknowledgements: The results have been acquired as part of the project “BURDEN 2020 – The Burden of disease in Germany at the national and regional level”; funded by the innovation fund of the Federal Joint Committee (project number 01VSF17007). We would like to thank the BURDEN study group for their support (BURDEN 2020 study group: Alexander Rommel, Elena von der Lippe, Annelene Wengler, Michael Porst, Aline Anton, Janko Leddin, Thomas Ziese (Robert Koch Institute); Helmut Schröder, Katrin Schüssel, Gabriela Brückner, Jan Breitkreuz (AOK Scientific Institute); Dietrich Plaß, Heike Gruhl (German Environment Agency)).

**References**

1. Porst M, von der Lippe E, Leddin J, Anton A, Wengler A, Breitkreuz J, et al. The burden of disease in Germany at the national and regional level. Dtsch Arztebl International. 2022;119(46):785-92.
2. Wengler A, Rommel A, Plaß D, Gruhl H, Leddin J, Ziese T, et al. Years of Life Lost to Death. Dtsch Arztebl International. 2021;118(9):137-44.

## A19 Changes in health situation for Turkey from 1990 to 2019: Based on the Global Burden of Disease Study 2019

### Vahit Yigit, Arzu Yigit

#### Süleyman Demirel University, Department of Health Management, Faculty of Economics and Administrative Sciences, Isparta, Turkey

##### **Correspondence:** Vahit Yigit (yigitv@hotmail.com)

*Archives of Public Health 2023*, **81(Suppl 2):**A19

**Background**

The burden of disease studies is analyzed in many countries to assess important health problems. The economic, political, and social changes experienced in recent years have affected the health status of the population in Turkey as well as in many other countries. As a result of the health policies implemented from 1990 to 2019 in Turkey, there has been an epidemiological transition that significantly improves health indicators. This research aims to compare the burden of diseases in Turkey from 1990 to 2019.

**Methods**

The burden of disease was calculated for the year 1990-2019 as years of life lost (YLLs), years lived with disability (YLDs), disability adjusted life years (DALYs), and the contribution of major risk factors to DALYs in Turkey. We used the data from the Global Burden of Disease (GBD) 2019 study [1, 2]. The GBD project is an international epidemiological project. All GBD methods, data, codes, and estimates are publicly available through interactive and visualization tools.

**Results**

While YLLs showed a great decrease of 58.4% (95% UI: 50.9–64.5) between 1990 and 2019 in Turkey, a very low decrease was observed in YLDs with 1.5% (95% UI: 0.99-3.86). DALY, which is the sum of YLL and YLD, decreased by 43.9% (95% UI: 37.5-49.7). In terms of the YLLs due to premature death in Turkey, ischemic heart disease, cerebrovascular disease, and congenital anomalies were the highest-ranking causes in 2019. The leading causes of YLDs in Turkey are major depressive disorder, low back pain, anxiety disorders, iron-deficiency anemia, and neck pain in 2019. The top causes of DALYs in 2019 were ischemic heart disease, neonatal disorders, stroke, and major depressive disorder in Turkey. The risk factors that account for the most disease burden in Turkey are tobacco, high body-mass index, and high blood pressure. The life expectancy at birth in Turkey has gradually increased since 1990 and reached 75.9 years for men and 81.3 for women in 2019. Child mortality in Turkey has gradually decreased since 1990 and reached 12.4 for under-1 and 15.4 for under-5 in 2019.

**Conclusions**

Despite significant progress in many communicable diseases and infant deaths between 1990 and 2019 in Turkey, non-communicable diseases continue to be the leading causes of death. A cost-effective, accessible, and sustainable health system and public health planning are needed in Turkey, taking into account major demographic changes. Requires a health policy and strategy to overcome financial and technical barriers to transferring health risk factors and their determinants to health information systems.

**References**

1. Institute for Health Metrics and Evaluation. Global Health Data Exchange. GBD Results Tool. http://ghdx.healthdata.org/gbd-results-tool (Accessed 15 May 2022).
2. Abbafati C, Abbas KM, Abbasi-Kangevari M, et al. Global burden of 369 diseases and injuries in 204 countries and territories, 1990–2019: a systematic analysis for the Global Burden of Disease Study 2019. Lancet. 2020;396(10258):1204-1222. doi:10.1016/S0140-6736(20)30925-9.

## A20 Burden of disease in Korea, 2008-2019: results from the national burden of disease study

### Seok-Jun Yoon^1^, Yoon-Sun Jung^2^, Young-Eun Kim^3^, Minsu Ock^4^

#### ^1^Department of Preventive Medicine, Korea University College of Medicine, Seoul, Republic of Korea; ^2^Institute for Future Public Health, Korea University, Seoul, Republic of Korea; ^3^Department of Big Data Strategy, National Health Insurance Service, Wonju, Republic of Korea; ^4^Department of Preventive Medicine, University of Ulsan College of Medicine, Ulsan, Republic of Korea

##### **Correspondence:** Seok-Jun Yoon (yoonsj02@korea.ac.kr)

*Archives of Public Health 2023*, **81(Suppl 2):**A20

**Background**

In South Korea, efforts have been made to measure the burden of disease using domestic data sources, and the scope of measurement has been expanded to identify health level by demographic factors such as region and income level. This study aimed to elucidate the trends and differences in the burden of disease in South Korea during 2008-2019 by updating the previous studies.

**Methods**

We calculated the YLL, YLD, and DALY for Koreans from 2008 to 2019 using an incidence-based approach. For YLL and YLD measurement, we used the data on the cause of death from Statistics Korea and National Health Insurance Service claims data by year. DALY calculation results were expressed in units per 100,000 population by disease, sex, region, and income level. For regional classification, 250 Korean municipal-level administrative districts were selected. The insurance premium was adopted to calculate the income as a proxy indicator. We used an equalized annual household income based on insurance premium and divided into quintiles by year and sex.

**Results**

From 2008 to 2019, the DALY rate increased 19.3% (from 22,361 to 26,685). Since 2017, the burden of disease among men has been higher than that among women. Diabetes mellitus, low back pain, and ischemic heart disease were ranked high in the burden of disease. In the past 11 years, the DALY rate of Alzheimer’s disease and other dementias increased by 303.3%, and glaucoma and benign prostatic hyperplasia also showed a steep increase. There was a 1.8-fold difference between the DALY rates of the highest and lowest regions. The burden of disease tends to decrease as the income level increases. DALY rate of 32,863 in the lowest income level (Q1), was 1.36 times higher than that of the highest income level (24,164 per 100,000 in Q5).

**Conclusions**

Estimating the difference and characteristics in the burden of disease within a country can help understand the health equity and the scale of the problem. The findings from this study can provide valuable and quantitative data for setting national health policy goals and prioritization and evaluation of health policies tailored to the characteristics of the burden of disease in the future. It is necessary to identify rapidly increasing disease by continuously monitoring and to prepare countermeasures.

